# Accuracy of MRI in Detecting 1p/19q Co-deletion Status of Gliomas: A Single-Center Retrospective Study

**DOI:** 10.7759/cureus.51863

**Published:** 2024-01-08

**Authors:** Adnan Naeem, Namrah Aziz, Manal Nasir, Hussain Sohail Rangwala, Hareer Fatima, Fatima Mubarak

**Affiliations:** 1 Department of Radiology, Aga Khan University Hospital, Karachi, PAK; 2 Department of Radiology, Aga Khan Health Service, Karachi, PAK; 3 Department of Medicine, Jinnah Sindh Medical University, Karachi, PAK

**Keywords:** idh-wildtype, idh, 1p/19q co-deletion, mri, oligodendrogliomas, central nervous system tumors

## Abstract

Background

Oligodendrogliomas, rare brain tumors in the frontal lobe's white matter, are reshaped by molecular markers like isocitrate dehydrogenase mutations and 1p/19q co-deletion, influencing treatment outcomes. Despite the initial indolence, these tumors pose a significant risk, with a median survival of 10-12 years. Non-invasive alternatives, such as magnetic resonance imaging (MRI) for assessing T2-fluid-attenuated inversion recovery (FLAIR) mismatch and calcifications, provide insights into molecular subtypes and aid prognosis. Our study explored these features to predict the oligodendroglioma status and refine patient management to improve outcomes.

Methods

In this retrospective study, patient data identified patients with suspected central nervous system tumors undergoing MRI, revealing low-grade gliomas. Surgical biopsy and 1p/19q fluorescence in situ hybridization confirmed the co-deletion status. MRI was used to assess various morphological features. Statistical analyses included x2 tests, Fisher’s exact tests, Kruskal-Wallis tests, and binary logistic regression models, with significance set at p < 0.05.

Results

Seventy-three patients (median age, 37 years) were stratified according to 1p/19q co-deletion. Most (61.6%) were 18-40 years old and mostly male (67.1%). Co-deletion cases, primarily frontal lobe lesions (67.6%), were unilateral (88.2%), with 55.9% non-circumscribed margins and 58.8% ill-defined contours. Smooth contrast enhancement and no necrosis were observed in 48.1% of 1p/19q co-deletion cases. Logistic regression analysis showed a significant association between ill-defined/irregular contours and 1p/19q co-deletion. Fisher's exact test confirmed this but raised concerns about the small sample size influencing the conclusions.

Conclusions

This study established a significant link between glioma tumor contour characteristics, particularly irregular and ill-defined contours, and the likelihood of 1p/19q co-deletion. Our findings underscore the clinical relevance of using tumor contours in treatment decisions and prognosis assessments.

## Introduction

Oligodendrogliomas are rare, ranking as the third most common primary brain tumor, and account for approximately 5% of all primary intracranial tumors. It is most commonly observed in cerebral white matter. Although it predominantly originates from the frontal lobe, it is also known to arise from the parietal or occipital lobes. A slow-growing tumor with diffuse infiltration, oligodendrogliomas are primarily located in the cortical-subcortical region and extend into the surrounding white matter [1].

As per the 2021 World Health Organization guidelines, central nervous system (CNS) tumors are now classified by incorporating both the histological data (phenotype) and molecular parameters (genotype). Among the crucial molecular markers are the isocitrate dehydrogenase (IDH) enzyme mutation status and the co-deletion of chromosome arms 1p and 19q (1p/19q). The former classification of adult-type diffuse gliomas has now been restructured into three distinct categories: astrocytoma, characterized by mutations in IDH without 1p/19q co-deletion; oligodendroglioma, with IDH mutations and 1p/19q co-deletion; and glioblastoma, IDH wild type [2,3]. These markers carry significant prognostic value, thus greatly influencing the treatment and patient outcomes. Epigenetic alterations and hypermethylation of the genome associated with the 1p/19q mutation result in a unique biological characteristic that correlates with enhanced chances of survival [1]. Studies have shown the presence of 1p/19q co-deletion is a strong indicator of better survival rates and a longer time-to-malignant transformation, regardless of tumor grade [4,5].

Though oligodendroglioma has an initial indolent disease course, they are almost fatal, as the median survival time of these patients is 10-12 years. To accurately predict the presence of oligodendrogliomas, it is essential to identify both an IDH1/IDH2 mutation and a 1p/19q co-deletion. This predictive approach can serve as a valuable prognostic tool [1]. The molecular status of gliomas is typically determined using tissue samples obtained through biopsy or resection procedures. These methods carry risks and may not provide a complete representation of the spatial variability within tumors. Additionally, in low-resource settings, however, DNA analysis methods are inaccessible. In light of these limitations, imaging modalities like MRI have gained popularity as a promising alternative, offering the advantage of determining the molecular status of gliomas non-invasively [6]. Several studies have demonstrated that the absence of T2-fluid-attenuated inversion recovery (FLAIR) mismatch is a highly specific indicator for the IDH-mutated and 1p/19q co-deleted molecular subtype in low-grade gliomas (LGGs) [7]. Lasocki et al. further revealed that a T2-FLAIR mismatch exceeding 50% strongly predicts a non-co-deleted tumor [8]. Recent meta-analyses indicate that this MRI feature serves as a specific biomarker for diagnosing IDH mutant non-co-deleted LGGs with a specificity of 99% [9]. However, it occasionally yields false positives in other tumor types and has notable false-negative results. Combining it with additional imaging parameters may enhance its diagnostic accuracy [10].

The presence of calcifications is strongly indicative of a 1p/19q co-deletion status [11]. As demonstrated in the study conducted by Yamauchi et al., tumors with IDH mutation and 1p/19q co-deletion frequently exhibit calcifications, especially in the frontal lobe [12]. The presence of calcifications, coupled with a larger preoperative tumor volume, could also serve as preoperative indicators to distinguish between malignancy grades II and III in oligodendrogliomas exhibiting IDH mutation and 1p/19q co-deletion [13]. In T2-weighted images, the presence of heterogeneous signal intensity is markedly more prevalent in IDH mutant/co-deleted gliomas compared to both IDH mutant and IDH wild-type tumors [12]. van Lent et al. demonstrated that within 1p/19q-co-deleted gliomas, the parameter of heterogeneity exhibited a sensitivity of 96%, while calcification displayed a specificity of 88.1% [14]. Gliomas with 1p/19q co-deletion are characterized by poorly defined borders but show cysts and edema more frequently [14,15]. Indeed, distinct tumor margins have been consistently associated with non-co-deleted LGGs in various investigations [11]. In light of the substantial impact of molecular markers, particularly 1p/19q co-deletion, on the prognosis and management of oligodendrogliomas, as well as the limitations of invasive testing, our study aims to explore the use of these MRI features in non-invasively predicting the molecular status of oligodendrogliomas. This can allow for precise patient management and improved outcomes in this challenging disease.

## Materials and methods

Study design, clinical setting, study duration, and sampling technique

This cross-sectional, retrospective validation study was conducted at the Department of Radiology and Department of Pathology in Aga Khan University Hospital, Karachi, over a two-year period from 2019 to 2021. Institutional review board approval was obtained from the Ethics Committee of Aga Khan University for waived consent (approval number: 2019-1055-2886). The sampling technique employed was non-probability retrospective sampling.

Sample size calculation

According to a study by Johnson et al. [16], genetically defined oligodendroglioma (1p/19q co-deleted) shows an indistinct tumor border on MRI with a sensitivity of 92% and specificity of 55%, and the expected prevalence of oligodendroglioma in LGGs according to this study is 61%. Using the above values, the sample size was calculated using the WHO sample size calculator with a precision of 8% and a confidence interval of 95%, and the estimated sample size was calculated to be 383. Since the calculated sample of 383 is not achievable because the disease is rare, only data from 73 cases were obtained in the span of two years and included in this study.

Inclusion and exclusion criteria

The inclusion criteria for this study were patients referred to the Radiology Department of Aga Khan University Hospital, specifically those who had undergone an MRI for LGG. Confirmation of the 1p/19q co-deletion status was a crucial requirement, which was determined through molecular genetic testing following a biopsy of the lesion. Notably, the study welcomed participants of all ages and sexes, aiming to ensure a comprehensive and diverse representation of individuals with LGGs. The exclusion criteria for this study included patients with CNS tumors other than LGGs (oligodendroglioma and astrocytoma). Additionally, individuals with a previously diagnosed oligodendroglioma who presented for follow-up examinations were excluded. Surgical interventions performed before the study and patients who had undergone radiotherapy or chemotherapy were also excluded from the study. These criteria were established to specifically focus the study on patients with LGGs who met certain conditions, ensuring a more homogeneous and targeted study population.

Operational definition

In this study, several operational definitions were established for clarity and precision. Suspected patients were identified based on a prolonged history of headaches that were either not relieved or temporarily relieved by analgesics. The category of LGGs encompasses grade II and grade III oligodendrogliomas, along with grade II and grade III astrocytomas. The term "1p/19q codeletion" refers to the combined loss of the short arm of chromosome 1 and the long arm of chromosome 19. A glioma with a 1p/19q co-deletion was specifically termed a genetically defined oligodendroglioma. In contrast, genetically defined astrocytomas were defined as gliomas with intact 1p/19q status. These operational definitions are crucial to ensure consistency and precision in characterizing suspected patients and classifying gliomas based on their genetic features.

Patient identification and data collection

In this study, patient data were utilized to identify eligible participants who met the inclusion criteria, consisting of individuals referred to the Radiology Department at Aga Khan University Hospital with a clinical suspicion of CNS tumors. These participants subsequently underwent MRI head scans, which revealed LGGs. The morphological features considered for tumor description on MRI encompassed various aspects, including tumor location, unilateral/bilateral involvement, margins on T1 and T2 images (circumscribed, partially circumscribed, and non-circumscribed), contour on T2 images (smooth, irregular, and ill-defined), homogeneity (homogeneous or heterogeneous), contrast enhancement (heterogeneous, smooth, and no enhancement), presence of necrotic or cystic changes, paramagnetic susceptibility (evaluated with susceptibility-weighted imaging (SWI) when applicable), diffusion restriction (mean apparent diffusion coefficient (ADC) value), and the degree of tumor margin circumscription (complete, partial, and lack thereof). These parameters were systematically evaluated to provide a comprehensive description of LGGs identified using MRI scans.

Statistical analyses

We conducted a comprehensive examination of the relationship between 1p/19q co-deletion status and the observed imaging characteristics. For comparisons between different groups, we utilized x2 tests or Fisher's exact tests (as applicable) for nominal variables and the Kruskal-Wallis test for continuous or ordinal variables. To assess the correlation between 1p/19q co-deletion, indicative of genetic oligodendrogliomas, and imaging features, we employed binary logistic regression models. Additionally, we reported odds ratios, 95% confidence intervals, and Nagelkerke pseudo R2 to describe our findings succinctly. Throughout our analysis, a significance level of p < 0.05 was established, rendering results statistically significant in all tests.

## Results

A total of 73 patients were included in the study. The median age of the patients was 37 years. Table 1 shows the baseline demographics and MRI features of the study population stratified by 1p/19q co-deletion status. A higher frequency of patients (61.6%) were in the age range of 18-40 years as compared to 31.5% in the age range of 40-60 years and 6.8% above the age of 60 years. Male sex was predominant in the present study, comprising 49 (67.1%) cases as compared to female sex, consisting of 24 (32.9%) cases.

**Table 1:** Baseline characteristics and MRI features stratified by 1p/19q co-deletion status. * Reported with median and interquartile range; percentages are presented in columns.

Variable	Total (N = 73)	1p/19q co-deletion absent (N = 39)	Co-deletion present (N = 34)
Age/years			
18 to 40	45 (61.6%)	25 (64.1%)	20 (58.8%)
40 to 60	23 (31.5%)	11 (28.2%)	12 (35.3%)
>60	5 (6.8%)	3 (7.7%)	2 (5.9%)
Age/years*	37	35 (20.0)	38 (13.5)
Gender			
Male	49 (67.1%)	27 (69.2%)	22 (64.7%)
Female	24 (32.9%)	12 (30.8%)	12 (35.3%)
Laterality			
Unilateral	65 (89%)	35 (89.7%)	30 (88.2%)
Bilateral	8 (11%)	4 (10.3%)	4 (11.8%)
Location of lesion			
Temporal	20 (27.4%)	13 (33.3%)	7 (20.6%)
Frontal	43 (58.9%)	20 (51.3%)	23 (67.6%)
Parietal	10 (13.7%)	6 (15.4%)	4 (11.8%)
Margins			
Non-circumscribed	32 (44.4%)	13 (34.2%)	19 (55.9%)
Partially circumscribed	20 (27.8%)	13 (34.2%)	7 (20.6%)
Circumscribed	20 (27.8%)	12 (31.6%)	8 (23.5%)
Contour			
Ill-defined	29 (39.7%)	9 (23.1%)	20 (58.8%)
Irregular	40 (54.8%)	26 (66.7%)	14 (41.2%)
Smooth	4 (5.5%)	4 (10.3%)	0 (0.0%)
Homogenous			
Homogenous	30 (46.9%)	15 (38.5%)	15 (60.0%)
Non-homogenous	34 (53.1%)	24 (61.5%)	10 (40.0%)
Cystic/necrosis			
Absent	40 (54.8%)	18 (46.2%)	22 (64.7%)
Present	33 (45.2%)	21 (53.8%)	12 (35.3%)
Contrast enhancement			
Absent	22 (38.6%)	9 (30.0%)	13 (48.1%)
Heterogeneous	5 (8.8%)	4 (13.3%)	1 (3.7%)
Smooth	30 (52.6%)	17 (56.7%)	13 (48.1%)

Patients with 1p/19q co-deletion oligodendroglioma were predominantly male (64.7%), with a median age of 38 years. Oligodendroglioma lesions may present at various anatomical positions and distribution shows that the majority had a lesion at the frontal lobe (67.6%) while 20.6% and 11.8% had lesions in the temporal and parietal lobes, respectively. The lesions were mostly unilateral (88.2%). Approximately half of the 1p/19q co-deletion patients had non-circumscribed margins (55.9%) and ill-defined contours (58.8%). Homogeneity was present in 60% of the cases, while cystic changes or necrosis were predominantly absent and were observed in only 35.3% of the cases. Contrast enhancement appeared smooth and was absent in an equal number of patients (48.1%) with 1p/19q co-deletion (Table 1).

On applying binary logistic regression (Table 2), the overall model was significant for ill-defined and irregular contours (p = 0.006) compared to smooth contour to ill-defined (p = 0.9) (ill-defined contour was taken as the reference category). The odds of co-deletion were 0.24 times less in irregular contour than in ill-defined contour with moderate negative relationship (p = 0.006, OR = 0.24, Nagelkerke pseudo R2 = 0.22, CI = 0.08-0.67).

**Table 2:** Binary logistic regression analyses for MRI features with 1p/19q co-deletion as the main explanatory covariate.

Variable	Odds ratio	P-value
Age/years	1.364 (0.50-3.73)	0.546
18 to 40		
40 to 60		
>60		
Gender	0.815 (0.31-2.17)	0.682
Male		
Female		
Laterality	1.167 (0.28-5.07)	0.837
Unilateral		
Bilateral		
Location of lesion	2.136 (0.71-6.40)	0.175
Temporal		
Frontal		
Parietal		
Margins	0.368 (0.12-1.17)	0.091
Non-circumscribed		
Partially circumscribed		
Circumscribed		
Contour	0.242 (0.08-0.67)	0.006
Ill-defined		
Irregular smooth		
Homogenous	0.417 (0.15-1.17)	0.095
Homogenous		
Non-homogenous		
Cystic/necrosis	0.468 (0.18-1.20)	0.114
Absent		
Present		
Contrast enhancement	0.173 (0.01-1.82)	0.144
Absent		
Heterogeneous smooth		

The data were reanalyzed using Fisher’s exact test for tissue contour to co-deletion. The data showed a significant difference (association) between co-deletions and irregular and ill-defined contours (p = 0.007). Smooth contour and ill-defined contour showed significant differences (p = 0.017) in co-deletion, and the sample size was small for smooth contour (N = 4). This might lead to alpha errors and, thus, the inability to conclude the significance. The investigation into the paramagnetic susceptibility effect through SWI and the measurement of mean, minimum, and maximum ADC values for each tumor did not yield statistically significant results. The data analysis suggests that there were no significant associations or differences in the evaluated parameters within the scope of the study.

**Figure 1:**
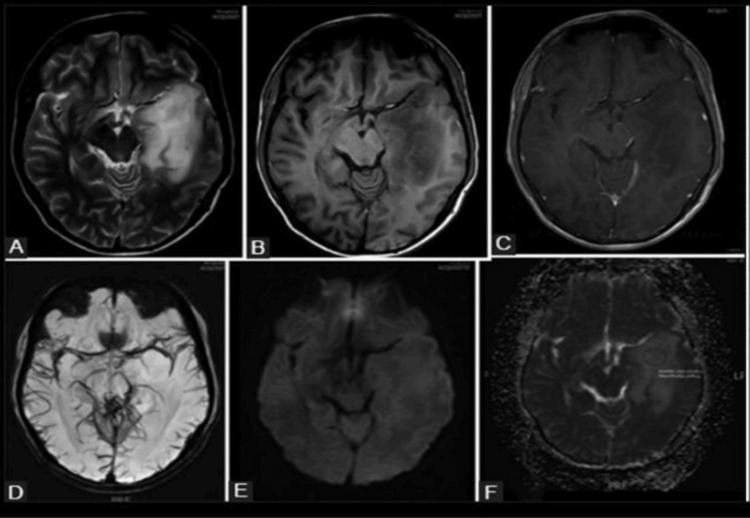
MRI of a patient with low-grade glioma without 1p/19q co-deletion (grade II astrocytoma). (A) Axial T2-weighted imaging (T2WI) provides detailed visualization of tissue characteristics and structural features. (B) Axial T1-weighted imaging (TIWI) captures the lesion's signal intensity on T1-weighted images. (C) Axial TIWI post-contrast highlights any contrast enhancement within the lesion, aiding in the identification of abnormal vascularity. (D) Susceptibility-weighted imaging (SWI) depicts susceptibility changes, contributing to the assessment of microhemorrhages and venous structures. (E) Diffusion-weighted imaging (DWI) is sensitive to the random motion of water molecules, facilitating the evaluation of tissue cellularity. (F) Apparent diffusion coefficient (ADC) mapping derives quantitative information about water diffusion, assisting in characterizing tissue properties. The representative images collectively illustrate a lesion situated in the left temporal lobe. Key observations include circumscribed margins, homogeneity on both T1 and T2-weighted images, absence of enhancement on post-contrast imaging, and the lack of internal cystic components. Notably, the lesion exhibits high mean ADC values on the ADC map, suggesting increased water diffusivity.

In Figure 1, an array of MRI sequences, denoted as A to F, was employed to illustrate the distinct characteristics of a low-grade glioma without 1p/19q co-deletion, specifically identified as a grade II astrocytoma. The lesion, situated in the left temporal lobe, exhibits well-defined margins, homogeneity on T1 and T2-weighted images, and a lack of enhancement or internal cystic components following contrast administration. Notably, the diffusion-weighted imaging (DWI) and the corresponding ADC mapping demonstrate elevated mean ADC values within the lesion, indicative of heightened water diffusivity.

**Figure 2:**
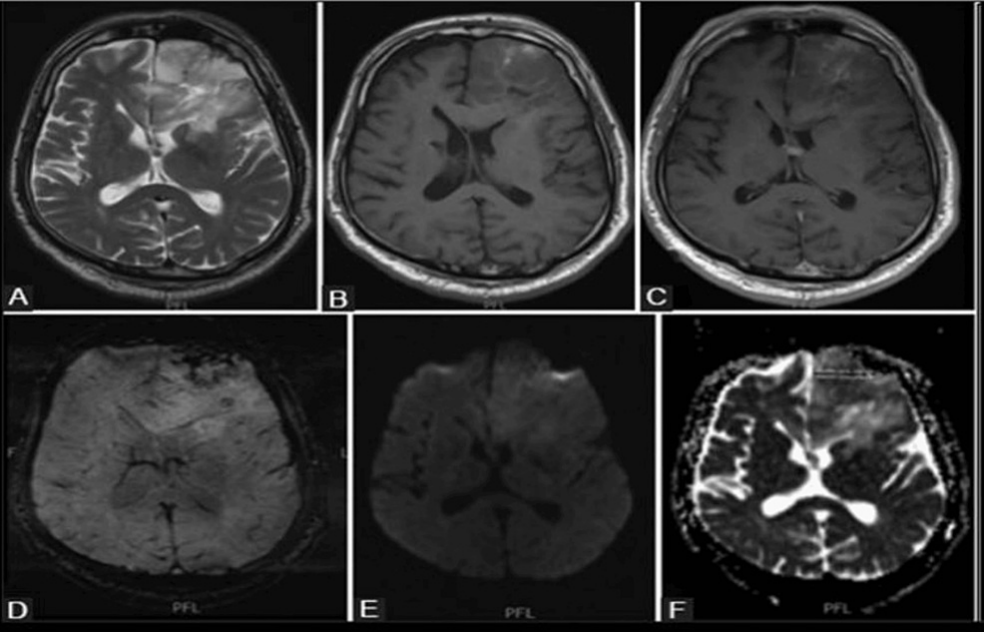
MRI of 1p/19q co-deleted low-grade glioma (oligodendroglioma). (A) Axial T2-weighted imaging (T2WI). T2WI is employed to visualize the anatomical structures with high water content. In the presented MRI, this sequence reveals the lesion in the left frontal lobe of the brain. (B) Axial T1-weighted imaging (TIWI). TIWI is utilized to highlight differences in tissue composition. The axial T1-weighted image in this figure provides additional details about the lesion's characteristics, helping in its assessment and classification. (C) Axial TIWI post-contrast administration). Post-contrast TIWI enhances the visibility of contrast-enhancing features, such as blood-brain barrier disruption. The axial post-contrast T1-weighted image in the figure allows for the identification of specific characteristics associated with the lesion, contributing to a more nuanced diagnosis. (D) Susceptibility-weighted imaging (SWI). SWI is sensitive to magnetic susceptibility differences between tissues, highlighting areas of blood or mineral deposition. The SWI sequence aids in detecting microhemorrhages or calcifications within the tumor, offering additional information for a comprehensive assessment. (E) Diffusion-weighted imaging (DWI). DWI assesses the movement of water molecules in tissues. In the presented MRI, the DWI sequence assists in evaluating the lesion's cellular density and organization. (F) Apparent diffusion coefficient (ADC) map. The ADC map is derived from DWI and provides quantitative information about the restriction of water diffusion within tissues. In Figure 2, the ADC map is utilized to calculate and visualize mean ADC values. A low mean ADC value is indicative of restricted diffusion, which can be associated with increased cellularity in the lesion.

Figure 2 displays MRI findings of a low-grade glioma, specifically an oligodendroglioma, characterized by the 1p/19q co-deletion. The various imaging sequences provide comprehensive insights into the tumor's characteristics, aiding in its diagnosis and characterization.

## Discussion

This comprehensive study delves into the intricate interplay between clinical and radiological factors and explores their connection with the presence of 1p/19q co-deletion in gliomas. By providing nuanced insights into the predictive power of various tumor characteristics, this study significantly contributes to our evolving understanding of the complex relationship between tumor morphology and genetic alterations in gliomas.

Among the diverse factors investigated, tumor contour has emerged as a particularly promising predictor, demonstrating potential implications for both diagnostic and therapeutic considerations. Specifically, the study revealed that irregular or ill-defined contours exhibit a discernible association with higher rates of 1p/19q co-deletion, suggesting a pivotal role of these morphological features in the identification of oligodendrogliomas with specific genetic profiles [11,12]. To underscore the significance of tumor contours, quantitative analysis was employed, highlighting the substantial difference in odds ratios between irregular and ill-defined contours. Tumors with irregular contours demonstrate markedly lower odds of harboring 1p/19q co-deletions compared to those with ill-defined contours, emphasizing the potential of morphological characteristics, particularly tumor contour, as valuable indicators of underlying genetic alterations [13,14].

The robustness of the association between tumor contour and 1p/19q co-deletion was further confirmed using a logistic regression model, reinforcing the overall significance of tumor contour as a potential visual cue for clinicians and radiologists in determining the genetic profile of gliomas. This methodological approach enhances the credibility of the study and emphasizes the reliability of the findings [17]. The discernible link between tumor contour and 1p/19q co-deletion is suggested to be rooted in the complex genetic pathways involved in glioma genesis. Genetic alterations such as 1p/19q co-deletion in oligodendrogliomas are known to influence tumor behavior and response to treatment. Therefore, the study calls for a deeper exploration of the molecular pathways associated with irregular contours to unveil specific molecular signatures contributing to the observed correlation [18,19]. Recent advancements in molecular biology techniques, such as single-cell sequencing and multi-omics, offer promising avenues for investigating heterogeneity within gliomas. The study that employed single-cell RNA sequencing has been used to unravel intratumoral heterogeneity in glioblastomas, revealing distinct molecular subpopulations. Integrating such high-resolution molecular data with radiological features, including tumor contours, could provide a more nuanced understanding of the genetic landscape associated with different morphological patterns [20,21]. The study employed statistical rigor through the application of Fisher's exact test, confirming a significant difference (p = 0.007) in co-deletion rates between irregular and ill-defined contours. This methodological approach not only strengthens the credibility of the study but also supports the assertion that irregular contours are less likely to result in 1p/19q co-deletion status in gliomas [22].

Understanding the genetic mechanism underlying the 1p/19q co-deletion in oligodendrogliomas is crucial. It involves a translocation event followed by preferential loss of the fusion chromosome, resulting in a whole-arm deletion of 1p and 19q. The putative tumor suppressor genes affected by 1p/19q co-deletion are believed to reside in regions 1p34-36 and 19q13, with a suspected role of reduced STATHMIN gene dosage in increased sensitivity to chemotherapy [20-23]. To further contextualize the findings, this study emphasizes the importance of considering existing literature. Previous studies have demonstrated a correlation between irregular tumor contours and specific genetic signatures in a cohort of glioblastomas, aligning with the current observations and suggesting potential generalizability across different glioma subtypes [22-24]. Additionally, a study that investigated the association between radiological features and molecular subtypes of gliomas provided further evidence of the relevance of tumor morphology in predicting genetic alterations. Their findings support the notion that specific radiological characteristics, including tumor contour, may serve as valuable noninvasive markers for the underlying genetic profiles in gliomas [25].

However, this study acknowledges its limitations in ensuring scientific rigor. While results related to tumor contours are robust, caution is warranted concerning the observed significant difference in co-deletion rates between smooth and ill-defined contours (p = 0.017). The small sample size (N = 4) in the smooth contour category raises concerns about result reliability, emphasizing the need for future research with larger and more diverse cohorts. A comprehensive representation of each contour category, particularly for smooth contours, is imperative to draw definitive conclusions regarding their association with 1p/19q co-deletion [7]. The clinical implications of these findings are substantial, resonating with the broader context of precision medicine in oncology. The identification of tumor contour as a non-invasive marker for predicting 1p/19q co-deletion status holds promise for guiding treatment decision-making, risk stratification, and prognosis assessment in glioma management. This aligns with the evolving paradigm of tailoring therapeutic approaches based on the underlying genetic makeup of tumors [23].

In the broader literature, the integration of radiological and genetic information for glioma characterization has gained significant attention. Studies have explored the synergistic role of radiogenomics, emphasizing the need for a holistic understanding of tumor biology [25,26]. Our findings contribute to this growing body of evidence, reinforcing the notion that radiological features, specifically tumor contours, can serve as valuable indicators of genetic alterations in gliomas [22-24]. Moreover, the link between tumor morphology and genetic characteristics has implications for prognosis and treatment response, demonstrating that certain genetic alterations, including 1p/19q co-deletion, are associated with an improved response to specific therapies in glioma patients. By identifying these genetic signatures through noninvasive radiological assessments, clinicians can tailor treatment strategies, potentially leading to enhanced therapeutic outcomes and improved patient survival rates [23-25].

The field of glioma research is rapidly evolving, with ongoing efforts to unravel the intricate complexities of tumor biology. Integrating radiological and genetic data provides a comprehensive approach to understanding glioma heterogeneity, guiding personalized treatment decisions, and improving patient outcomes [27,28]. As technological advances and larger datasets become available, future research endeavors can build upon our current findings, refine predictive models, and expand the applicability of noninvasive markers in glioma management. Although our study emphasizes the association between tumor contour and 1p/19q co-deletion, it is essential to acknowledge the dynamic nature of gliomas. Tumor evolution and clonal selection over time can influence both radiological features and genetic alterations. Longitudinal studies incorporating sequential radiological and genetic assessments would provide valuable insights into the temporal dynamics of these associations [21-25].

## Conclusions

In conclusion, this study establishes a significant association between tumor contour characteristics and the likelihood of 1p/19q co-deletion in gliomas. The findings, particularly regarding irregular and ill-defined contours, highlight the potential of these morphological features as noninvasive markers for predicting genetic alterations. While limitations, such as a small sample size in the smooth contour category, warrant caution, the study underscores the clinical relevance of utilizing tumor contour in treatment decision-making and prognosis assessment. Future research should focus on larger, more diverse cohorts to enhance generalizability and reliability, advancing the early detection of 1p/19q co-deletion status in LGGs and improving personalized therapeutic approaches.

## References

[1] Tork CA, Atkinson C (2023). Oligodendroglioma. https://www.ncbi.nlm.nih.gov/books/NBK559184/.

[2] Louis DN, Perry A, Wesseling P (2021). The 2021 WHO Classification of Tumors of the Central Nervous System: a summary. Neuro Oncol.

[3] Berger TR, Wen PY, Lang-Orsini M, Chukwueke UN (2022). World Health Organization 2021 classification of central nervous system tumors and implications for therapy for adult-type gliomas: a review. JAMA Oncol.

[4] Familiari P, Lapolla P, Picotti V (2023). Role of 1p/19q codeletion in diffuse low-grade glioma tumour prognosis. Anticancer Res.

[5] Smith JS, Perry A, Borell TJ (2000). Alterations of chromosome arms 1p and 19q as predictors of survival in oligodendrogliomas, astrocytomas, and mixed oligoastrocytomas. J Clin Oncol.

[6] Chakrabarty S, Sotiras A, Milchenko M, LaMontagne P, Hileman M, Marcus D (2021). MRI-based identification and classification of major intracranial tumor types by using a 3D convolutional neural network: a retrospective multi-institutional analysis. Radiol Artif Intell.

[7] Kihira S, Derakhshani A, Leung M (2023). Multi-parametric radiomic model to predict 1p/19q co-deletion in patients with IDH-1 mutant glioma: added value to the T2-FLAIR mismatch sign. Cancers (Basel).

[8] Lasocki A, Gaillard F, Gorelik A, Gonzales M (2018). MRI features can predict 1p/19q status in intracranial gliomas. AJNR Am J Neuroradiol.

[9] Han Z, Chen Q, Zhang L (2022). Radiogenomic association between the T2-FLAIR mismatch sign and IDH mutation status in adult patients with lower-grade gliomas: an updated systematic review and meta-analysis. Eur Radiol.

[10] Adamou A, Beltsios ET, Papanagiotou P (2021). The T2-FLAIR mismatch sign as an imaging indicator of IDH-mutant, 1p/19q non-codeleted lower grade gliomas: a systematic review and diagnostic accuracy meta-analysis. Diagnostics (Basel).

[11] Lasocki A, Buckland ME, Drummond KJ (2022). Conventional MRI features can predict the molecular subtype of adult grade 2-3 intracranial diffuse gliomas. Neuroradiology.

[12] Yamauchi T, Ohno M, Matsushita Y (2018). Radiological characteristics based on isocitrate dehydrogenase mutations and 1p/19q codeletion in grade II and III gliomas. Brain Tumor Pathol.

[13] Fukuya Y, Tamura M, Nitta M (2023). Tumor volume and calcifications as indicators for preoperative differentiation of grade II/III diffuse gliomas. J Neurooncol.

[14] van Lent DI, van Baarsen KM, Snijders TJ, Robe PA (2020). Radiological differences between subtypes of WHO 2016 grade II-III gliomas: a systematic review and meta-analysis. Neurooncol Adv.

[15] Rangwala BS, Shakil A, Mustafa MS, Rangwala HS, Fatima H, Siddiq MA (2023). Losartan and immune checkpoint inhibitors in glioblastoma: an appropriate substitute for steroids. [PREPRINT]. Ann Neurosci.

[16] Johnson DR, Diehn FE, Giannini C, Jenkins RB, Jenkins SM, Parney IF, Kaufmann TJ (2017). Genetically defined oligodendroglioma is characterized by indistinct tumor borders at MRI. AJNR Am J Neuroradiol.

[17] Wu A, Aldape K, Lang FF (2010). High rate of deletion of chromosomes 1p and 19q in insular oligodendroglial tumors. J Neurooncol.

[18] Gresner SM, Rieske P, Wozniak K (2007). Gliomas: association of histology and molecular genetic analysis of chromosomes 1p, 10q, and 19q. Acta Neurobiol Exp (Wars).

[19] Ohgaki H, Kleihues P (2005). Population-based studies on incidence, survival rates, and genetic alterations in astrocytic and oligodendroglial gliomas. J Neuropathol Exp Neurol.

[20] Nigro JM, Takahashi MA, Ginzinger DG, Law M, Passe S, Jenkins RB, Aldape K (2001). Detection of 1p and 19q loss in oligodendroglioma by quantitative microsatellite analysis, a real-time quantitative polymerase chain reaction assay. Am J Pathol.

[21] McDonald JM, See SJ, Tremont IW (2005). The prognostic impact of histology and 1p/19q status in anaplastic oligodendroglial tumors. Cancer.

[22] Gresner SM, Rieske P, Wozniak K (2006). Molecular analysis of chromosome 1, 10 and 19 abnormalities in human oligodendroglial tumors: relationship between frequency of LOH grade, age and gender. Clin Neuropathol.

[23] Fellah S, Caudal D, De Paula AM (2013). Multimodal MR imaging (diffusion, perfusion, and spectroscopy): is it possible to distinguish oligodendroglial tumor grade and 1p/19q codeletion in the pretherapeutic diagnosis?. AJNR Am J Neuroradiol.

[24] Ali LA, Usman KM, Fatima M (2021). Can apparent diffusion coefficient predict the grade, genotype, or proliferation index of oligodendrogliomas. Asian J Neurosurg.

[25] Ducray F, Idbaih A, de Reyniès A (2008). Anaplastic oligodendrogliomas with 1p19q codeletion have a proneural gene expression profile. Mol Cancer.

[26] Laghari AA, Khalid MU, Qadeer N, Shamim MS (2019). Prognostic value of 1p/19q chromosomal codeletion in patients with oligodendroglioma. J Pak Med Assoc.

[27] Lin Y, Xing Z, She D (2017). IDH mutant and 1p/19q co-deleted oligodendrogliomas: tumor grade stratification using diffusion-, susceptibility-, and perfusion-weighted MRI. Neuroradiology.

[28] Mughal ZUN, Fadlalla Ahmad TK, Haseeb A, Shafique MA, Abbas Ahmdon OE, Mahgoub AMA (2024). Dabrafenib and trametinib as a promising treatment option for pediatric population with low-grade gliomas that have BRAF V600E mutation; a breakthrough in the field of neuro-oncology. Int J Surg Glob Health.

